# Electroacupuncture ameliorates depression-like behaviors in rats with post-stroke depression by inhibiting ferroptosis in the prefrontal cortex

**DOI:** 10.3389/fnins.2024.1422638

**Published:** 2024-10-03

**Authors:** Jing Gao, Xiaolei Song, Yixuan Feng, Lihua Wu, Zhimin Ding, Shikui Qi, Mingyue Yu, Ruonan Wu, Xinyue Zheng, Yanyan Qin, Yuchuang Tang, Mengyu Wang, Xiaodong Feng, Qiongshuai Zhang

**Affiliations:** ^1^Department of Rehabilitation Center, The First Affiliated Hospital of Henan University of Chinese Medicine, Zhengzhou, China; ^2^Second Clinical Medical College, Guangzhou University of Chinese Medicine, Guangzhou, China; ^3^School of Rehabilitation Medicine, Henan University of Chinese Medicine, Zhengzhou, China; ^4^The First Affiliated Hospital of Zhengzhou University, Zhengzhou, China

**Keywords:** post-stroke depression, electroacupuncture, ferroptosis, depression-like behavior, iron accumulation

## Abstract

**Introduction:**

Post-stroke depression (PSD) is the most common complication following a stroke, significantly hindering recovery and rehabilitation in affected patients. Despite its prevalence, the pathogenesis of PSD remains poorly understood. Electroacupuncture (EA) has shown antidepressant effects, yet its neuroprotective properties are not well defined. Ferroptosis, a recently identified form of cell death, is implicated in the pathological processes of stroke and is associated with the development of depression-like behaviors. So we aimed to investigate whether PSD induces ferroptosis, identify potential therapeutic targets within these pathways, and elucidate the underlying mechanisms in this study.

**Methods:**

Male Sprague-Dawley rats were subjected to middle carotid artery occlusion and chronic unpredictable mild stress to model PSD. To explore the role of ferroptosis in the effects of EA, the ferroptosis inducer erastin was administered into the rats’ lateral ventricles, followed by 14 days of EA treatment, with sessions lasting 30 minutes per day. The Zea-Longa score was used to assess neurological deficits, while the sucrose preference test, elevated plus maze test, and open-field test were employed to evaluate depression-like behaviors in the rats. Hematoxylin-eosin, Nissl, and Perl’s staining were used to observe the morphological changes and iron deposition in the prefrontal neurons. Transmission electron microscopy provided detailed observations of mitochondrial morphological changes in neurons. We utilized activity assay kits, enzyme-linked immunosorbent assay (ELISA), and Western blotting to explore potential molecular mechanisms underlying the effects of EA.

**Results:**

EA can reduce neurological deficits and enhance the spontaneous activity and exploration behavior of rats. In addition, EA could inhibit prefrontal cortex neuronal ferroptosis by reducing iron deposition, decreasing lipid peroxidation, and enhancing antioxidation.

**Discussion:**

EA improved depression-like behaviors, mitigated mitochondrial damage, and inhibited ferroptosis in prefrontal cortex neurons. Notably, the administration of erastin further enhanced these effects. In conclusion, EA appears to improve PSD by inhibiting ferroptosis in the prefrontal cortex.

## 1 Introduction

Stroke is the second leading cause of death globally, accounting for 11.6% of all fatalities, and the third leading contributor to human disability (Kuriakose and Xiao, 2020). Among stroke survivors, post-stroke depression (PSD) is a prevalent complication, leading to emotional disorders and behavioral abnormalities. It is estimated that approximately one-third of stroke survivors develop depression (Tu et al., 2023). While previous studies have suggested that PSD is associated with both neurobiological and psychosocial factors, the precise pathophysiological mechanisms remain unclear (Kang et al., 2021).

Ferroptosis was first identified as a distinct form of programmed cell death by Dixon et al. (2012). It is characterized by excessive iron-dependent lipid peroxidation. In the presence of ferrous or lipoxygenase, highly expressed unsaturated fatty acids on the cell membrane are catalyzed, leading to lipid peroxidation, thereby inducing cell death. Additionally, a reduction in the activity of the intracellular antioxidant system, particularly the glutathione system, contributes to ferroptosis (Chen et al., 2021). This process plays an important role in the pathogenesis of stroke, and inhibition of ferroptosis helps to reduce brain damage (Wang L. et al., 2023). For instance, Chen et al. (2019) showed that ferrostatin-1 attenuates neuronal damage by reducing iron deposition in injured brain tissues. Moreover, an animal study on depression revealed abnormalities in iron metabolism, with excessive accumulation of lipid peroxides in the brains of mice subjected to chronic unpredictable mild stress (CUMS) (Cao et al., 2021). Therefore, inhibiting neuronal ferroptosis following a stroke may represent a potential treatment strategy for PSD.

Electroacupuncture (EA) is a traditional Chinese therapeutic method frequently used in the treatment of neuropsychiatric disorders. Clinical studies have demonstrated that EA is both safe and effective in treating PSD, as it not only alleviates depressive symptoms but also enhances the activities of daily living in PSD patients (Cai et al., 2022; Xie et al., 2022; Lam Ching et al., 2023). Furthermore, our previous research found that EA treatment could improve depression-like behavior in PSD model rats by activating adenosine 5’monophosphate-activated protein kinase (Ding et al., 2023). However, it remains unclear whether the inhibition of ferroptosis contributes to the protective effects of EA in treating PSD.

In this study, we aimed to investigate the potential antidepressant effects of EA in PSD rats. The underlying mechanisms were explored by measuring cerebral infarct size, depression-like behavior, iron deposition, oxidative stress, and expression of ferroptosis-related proteins. Our findings may provide valuable evidence supporting the effectiveness of EA in the treatment of PSD.

## 2 Materials and methods

### 2.1 Animals and ethics approval

A total of 165 male Sprague–Dawley rats, weighing 220–240 g, were obtained from Jinan Pengyue Experimental Animal Breeding Co. [license no. SCXK(Lu)20,190,003]. The rats were housed under controlled conditions: a 12-h light/dark cycle, a constant temperature of 22–25°C, 50% humidity, and *ad libitum* access to food and water. All experimental procedures adhered to the ARRIVE guidelines and were conducted in compliance with the U.K. Animals (Scientific Procedures) Act, 1986 as well as the National Research Council’s Guide for the Care and Use of Laboratory Animals. The experimental protocols were approved by the Ethics Committee of the Experimental Animal Centre of Henan University of Chinese Medicine (approval no. DWLL202206106).

### 2.2 Experimental design

**Figure 1:**
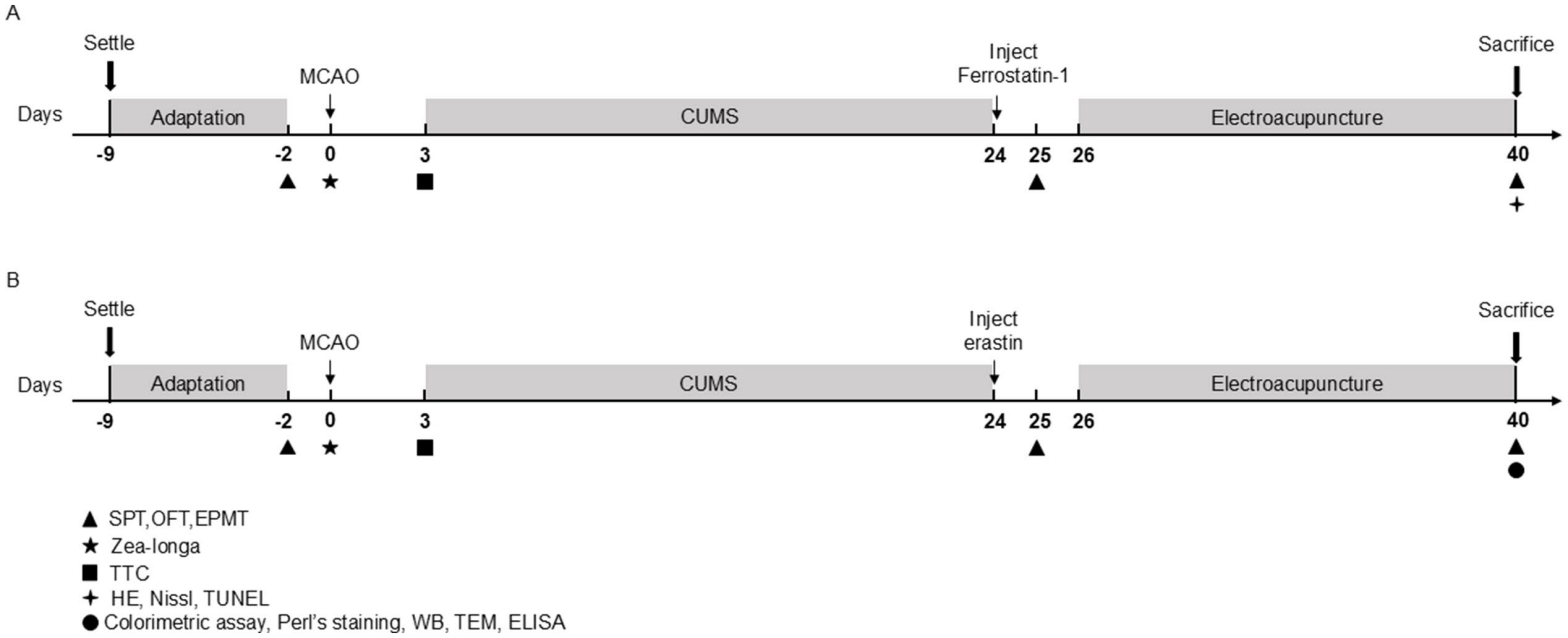
Schematic diagram showing the experimental design of the two study experiments. **(A)** Timeline of Experiment 1. **(B)** Timeline of Experiment 2. MCAO, middle cerebral artery occlusion; CUMS, chronic unpredictable mild stress; SPT, sucrose preference test; OFT, open-field test; EPMT, elevated plus maze test; TTC, 2,3,4-triphenytetrazolium-chloride; HE, hematoxylin–eosin staining; TUNEL, tdt-mediated dutp nick-end labeling; WB, Western blot; TEM, transmission electron microscope; ELISA, enzyme-linked immunosorbent assay.

The experiments in this study were divided into two parts (Figures 1A,B).

#### 2.2.1 Experiment 1

First, we evaluated the effects of EA treatment and ferrostain-1 (a specific inhibitor of ferroptosis) on the neurological functions and behavioral activities of PSD rats. Rats were randomly divided into four groups: the Sham group (*n* = 10), the PSD model group (PSD group) (*n* = 10), EA group (*n* = 10) and Ferrostain-1 group (Fer-1) (*n* = 9). Rats in this group underwent incision and suturing without the insertion of a thread plug. Rats in the PSD, EA and Fer-1 group underwent both middle cerebral artery occlusion (MCAO) and CUMS modeling. Rats in the EA group received EA treatment for 14 consecutive days following CUMS. Rats in the Fer-1 group were administered an intraperitoneal dose of Fer-1 (2.5 μmol/kg, GC10380, Glpbio, USA), a specific inhibitor of ferroptosis, once daily for 14 days, as previously reported (Ye et al., 2020). In Experiment 1, we conducted a series of assessments, including 2,3,5-triphenyltetrazolium chloride (TTC) staining, Zea-Longa scoring criteria, the sucrose preference test (SPT), the open-field test (OFT), the elevated plus maze test (EPMT), hematoxylin–eosin (HE) staining, Nissl staining, and TUNEL assay to evaluate the outcomes.

#### 2.2.2 Experiment 2

Next, to determine the role of ferroptosis in EA intervention in PSD rats, we randomly divided rats into five groups (*n* = 15 per group): the Sham group, PSD group, EA group, Erastin group, and Erastin+EA group. All rats except for the sham group received MCAO and CUMS modeling. Rats in the Erastin group were administered erastin (a ferroptosis inducer, IE0310, Solarbio, Beijing, China) after PSD modeling. In the Erastin+EA group, rats received both erastin administration and electroacupuncture stimulation, similar to the Erastin group. In Experiment 2, we conducted various assessments, including 2,3,5-triphenyltetrazolium chloride (TTC) staining, the Zea-Longa score, the sucrose preference test (SPT), the open-field test (OFT), the elevated plus maze test (EPMT), Perl’s staining, colorimetric assays, enzyme-linked immunosorbent assay (ELISA), transmission electron microscopy (TEM), and Western blotting (WB).

### 2.3 PSD modeling

The PSD model was established using MCAO and CUMS. The MCAO procedure was performed as previously described (Wang et al., 2021). Rats were anesthetized with 10% pentobarbital sodium (0.025–0.03 mg/g) administered via intraperitoneal injection. The right common carotid artery, external carotid artery, and internal carotid artery of rats were sequentially isolated. The external carotid artery was ligated, and the internal carotid artery was temporarily clamped. A monofilament nylon suture with a silicone tip (2636-50A4, Beijing Cinontech Co. Ltd., Beijing, China) was then inserted into the internal carotid artery through an incision made in the common carotid artery. The timing was calculated from the time that the suture was inserted 15–20 mm from the common carotid artery fork. The perfusion of the rats was restored after 2 h. During the operation, an electric blanket was used to keep the rats’ body temperature at about 37°C until they fully recovered from anesthesia. All sham-operated animals survived, but the overall mortality rate for MCAO was 17.1% (24/140) (Supplementary Table S1). Rats with Zea-Longa scores between 1 and 4 were considered to have successfully achieved the model.

CUMS procedures were conducted 2 days after MCAO in separate cages. Rats were subjected to a variable sequence of mild unpredictable stressors over 21 days, which included seven stressors: deprivation of food and water (24 h), wrap restraint (6 h), swimming in ice water at 4°C (5 min), exposure to a moist dwelling environment (24 h), horizontal vibration (5 min), and tail clamping (1 min, repeated three times).

### 2.4 Stereotactic injection

Rats were anesthetized with 10% pentobarbital sodium (0.025–0.03 mg/g), and their skulls were secured in a brain stereotaxic apparatus (RWD Life Science Co. Ltd., Shenzhen, China). A longitudinal incision was made along the scalp to expose the fontanel, which was used as the origin point for the coordinate system. The prefrontal cortex region (Bregma *x* = 1.5 mm, *y* = 1.1 mm, *z* = 4.5 mm) was localized and perforated according to the stereotactic atlas of the rat brain. The microsyringe tip was aligned perpendicularly to the brain surface, and a solution containing erastin (IE0310, Solarbio, Beijing, China) was injected slowly at a rate of 0.1 μL/min per side using an automatic syringe pump. After the injection, the incision was sutured, and the rats were administered an intraperitoneal injection of penicillin (15 mg/kg) to prevent infection.

### 2.5 EA treatment

**Figure 2:**
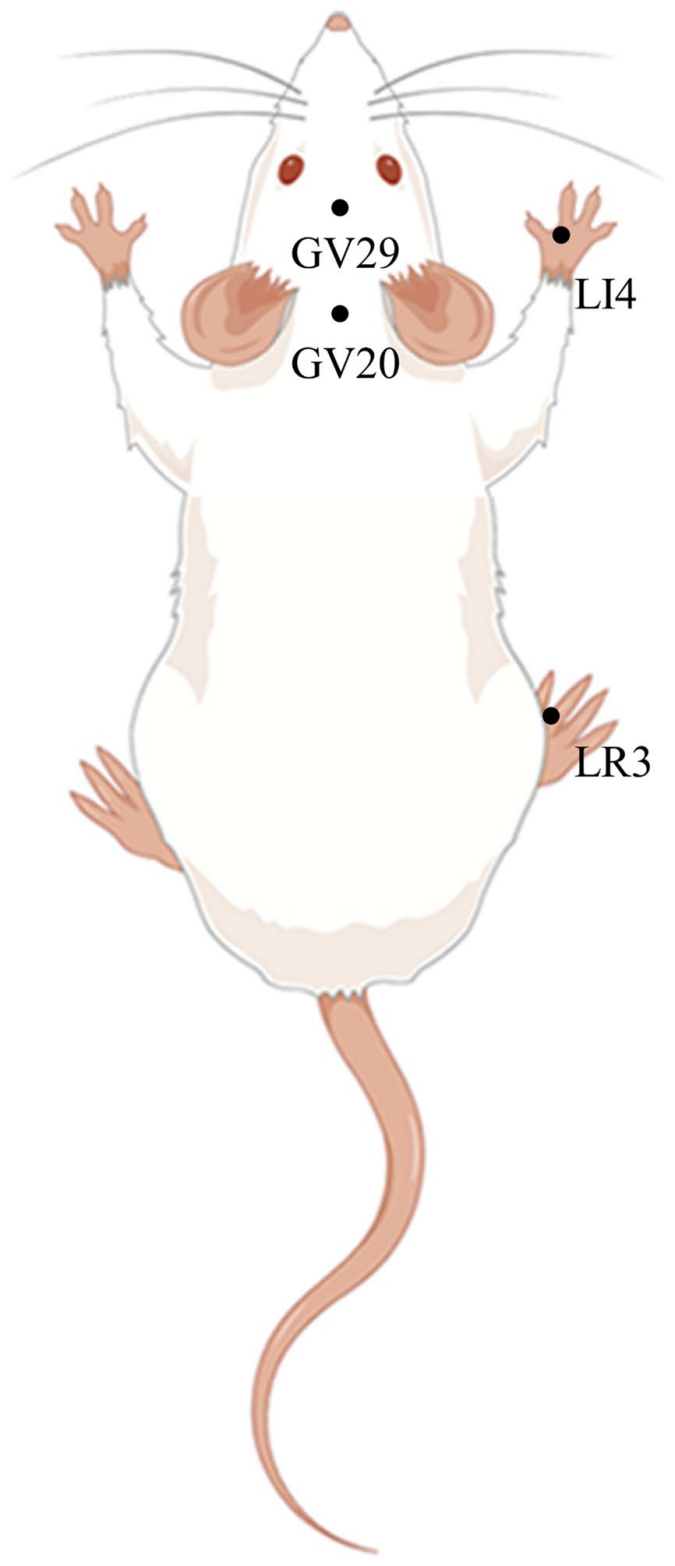
Schematic diagram of GV20, GV29, LI4, and LR3 in a rat (Created using Figdraw). Baihui (GV20) is located in the middle of the parietal bone of the rat; Yintang (GV29) is located between the eyes of the rat; Hegu (LI4) is located between the first and second metacarpal bones of the rat forelimb; Taichong (LR3) is located between the first and second metatarsal bones on the dorsum of the foot of rat hind limb.

Rats were secured on a rat fixator (Henan Zhike Hongrun Environmental Protection Technology Co., Zhengzhou, China). Sterilized, disposable stainless-steel needles (0.16 mm in diameter and 7 mm in length, Hwato Medical Instruments Co, Ltd., Suzhou, China), were inserted 2–3 mm at acupuncture points GV 20 (Baihui), GV 29 (Yintang), LI 4 (Hegu), and LR 3 (Taichong), as reported in previous studies (Gao J. et al., 2021). Then, the EA apparatus (Hwato, Suzhou Medical Supplies Factory Co. Ltd.) was activated to deliver EA stimulation to the LI 4 and LR 3 points (2/100 Hz, 1 mA, for 30 min). EA treatment commenced after PSD modeling in the EA group and Erastin+EA group, and was performed once for two weeks (Figure 2).

### 2.6 Zea-Longa scoring criteria

The Zea-Longa scoring criteria were used to assess neurological function 2 h after MCAO, with the following scoring system: 0: normal, no neurological injury symptoms; 1: mild neurological injury, indicated by contralateral forelimb adduction and flexion during tail lifting; 2: moderate neurological injury, characterized by lateral rotation during crawling; 3: severe neurological injury, with the rats falling to the opposite side when crawling; 4: no autonomous activity, accompanied by a disturbance of consciousness.

### 2.7 TTC

After the rats were anesthetized, the brain tissue was quickly removed and frozen at −20°C for 20 min. We used a razor blade to cut the prepared stained tissue into five thin slices. The slices were immersed in 1% TTC solution and incubated at 37°C away from light for 30 min. The tissue sections were occasionally turned to ensure full immersion in the staining solution. After staining, the cerebral infarction size was observed. Three rats from each group were used for TTC.

### 2.8 Behavioral tests

To assess depression-like behaviors, a series of behavioral tests, including the sucrose preference test (SPT), open-field test (OFT), and elevated plus maze test (EPMT), were conducted at three time points: 2 days before MCAO, and 25 and 40 days after MCAO.

#### 2.8.1 SPT

For adaptive training, rats were initially exposed to two bottles of 1% sucrose solution for 24 h, following which they were exposed to a bottle of 1% sucrose solution and a bottle of water for an additional 24 h. For the formal testing phase, after a 24-h period of water deprivation, rats were presented with two identical bottles, one filled with 1% sucrose solution and one with water. The positions of the bottles were randomly switched to avoid side preference. Sucrose and water consumption over the next 12 h were measured. The sucrose preference rate was calculated as previously described.

#### 2.8.2 OFT

Rats were placed in the center of the open square box (100 cm × 100 cm × 40 cm, RWD Life Science Co., Ltd.) and allowed to explore freely for 5 min. The bottom of the box was divided into three concentric rectangular areas, named the central, middle, and external areas. An infrared camera was employed to record the behaviors of rats. The total distance traveled and the time spent in the central area were analyzed by the software (SMART 3.0, RWD Life Science Co., Ltd., China). After the test, the box was sprayed with 75% ethanol to eliminate odors.

#### 2.8.3 EPMT

A cross-shaped maze (RWD Life Science Co., Ltd.) was placed on a 50-cm-high elevation. It consisted of two open arms (50 × 10 cm^2^), two closed arms (50 × 10 × 40 cm^3^), and a central area (10 × 10 cm^2^) connecting the open and closed arms. Before the experiment, the rats were acclimated in a behavioral laboratory. During the experiment, each rat was gently placed in the central area facing the open arm. A camera was installed above the maze and connected to a computer to track the rats and record the open arm time. After the test, the box was sprayed with 75% ethanol to eliminate odors.

### 2.9 HE, Nissl, and Perl’s staining

After 14 days EA treatment, rats were anesthetized and sacrificed. The brains were quickly removed and fixed in 4% formaldehyde for more than 24 h. The fixed tissue was sectioned into 4-μm-thick slices, dried with water, and dyed with HE, Nissl, and Perl’s staining according to the routine protocol. The neuron pathological changes in the prefrontal cortex were observed under a microscope (E100, Nikon, Japan). The number of rats in each group for HE, Nissl, and Perl’s staining was 3.

### 2.10 TUNEL staining

The brains were fixed in 10% formalin overnight at 4°C, washed with phosphate-buffered saline, and then embedded in paraffin. The tissues were sectioned into 5-μm-thick slices and then incubated with TUNEL staining reagents for TUNEL staining based on the manufacturer’s guidance (G1502-50 T, Servicebio, Wuhan, China). Images were captured by NIS-Element viewer software under the inverted microscope (E100, Nikon, Japan) and TUNEL-Positive rate in the prefrontal cortex were counted by ImageJ software. Three rats from each group were used for TUNEL staining.

### 2.11 Colorimetric assay

According to the instructions, colorimetric assay kits for ferrous iron (Fe2+) (E-BC-K773-M, Elabscience, Wuhan, China), total superoxide dismutase (T-SOD) (E-BC-K318-M, Elabscience), malondialdehyde (MDA) (E-BC-K025-M, Elabscience), glutathione peroxidase (GSH) (E-BC-K030-M, Elabscience), and catalase (CAT) (E-BC-K031-M, Elabscience) were used to detect the levels of Fe2+, T-SOD, MDA, GSH, and CAT in rat serum, respectively. Three rats from each group were used for colorimetric assay.

### 2.12 ELISA

The serum of rats in each group was diluted at a ratio of 1:9 (serum dilution buffer), and the procedure was strictly followed according to the manufacturer’s instructions (ml926281, Shanghai Enzyme-linked Biotechnology Co., Ltd., Shanghai, China). The optical density (OD) of each well was measured at 450 nm using the microplate reader (Multiskan SkyHigh, ThermoFisher scientific). After generating a standard curve, the serum reactive oxygen species (ROS) levels in the serum of rats were calculated based on the standard curve.

### 2.13 TEM

Fresh samples of prefrontal cortex (≤ 1 mm^3^) were collected and fixed with 2.5% phosphate-buffered glutaraldehyde at 4°C overnight and then post-fixed with 1% OsO4 for 2 h. Then, the samples were dehydrated in a graded series with ethanol and acetone and embedded in epoxy resin. Ultrathin sections (approximately 70-nm-thick) were cut using an ultramicrotome and stained with 2% uranyl acetate. Finally, the sections were viewed under a TEM (JEM-1400, Japan Electron Biotransmission Electron Microscope, Tokyo, Japan). Three rats in each group were used for TEM.

### 2.14 WB

The prefrontal cortices of rats were sectioned into appropriate sizes and weighed. The tissue samples were then homogenized in RIPA lysis buffer with grind beads and placed on ice for lysis. Protein concentrations were determined using the BCA Protein Assay Kit (PC0020, Solarbio). Finally, sample buffer was added according to the protein concentration of each sample, and the solution was boiled for 10 min. Proteins were first separated using a 10% SDS-PAGE gel at 80 V for 15 min, and then at 120 V for 120 min. After being transferred to a PVDF membrane, the specimens were blocked with 5% skim milk for 2.5 h and then incubated overnight at 4°C with primary antibodies against glyceraldehyde 3-phosphate dehydrogenase (GAPDH) (1:5000, 60,004-1-Ig, Proteintech, Wuhan, China), ferritin heavy chain (FTH-1) (1:2000, ab183781, Abcam), ferritin light chain (FTL) (1:2000, ab69090, Abcam), solute carrier family 7 member 11 (SLC7A11) (1:10000, ab175186, Abcam), glutathione peroxidase 4 (GPX4) (1:4000,67,763-1-lg, Proteintech), acyl-coa synthetase long chain family member 4 (ACSL4) (1:10000, ab155282, Abcam), and lysyloxidase (LOX) (1:10000, ab174316, Abcam). On the next day, the specimens were washed three times on the shaker with 1 × TBST for 10 min each time. Then, HRP-goat anti-rabbit (1:10000, SA00001-2, Proteintech) and HRP-goat anti-mouse IgG (1:10000, SA00001-1, Proteintech) were added and incubated at 4°C for 1 h. Finally, ECL Western Blotting Substrate (PE0010, Solarbio) was used for exposure, and Image J software was used to examine the relative protein level. Three rats in each group were used for WB.

### 2.15 Statistical analysis

All statistical analyses were performed using GraphPad Prism 8.0 (San Diego, CA, USA). This software was used to generate bar charts and line charts. Data were presented as mean ± standard error of the mean (*SEM*). Differences among groups were determined using one-way analysis of variance and followed by calculation of Tukey test when equal variances were assumed. Otherwise, a Dunnett’s T3 test was conducted. Kruskal-Wallis test was used to Zea-Longa score. *p* < 0.05 was considered statistically significant.

## 3 Results

### 3.1 EA treatment improved depression-like behavior in PSD rats

**Figure 3:**
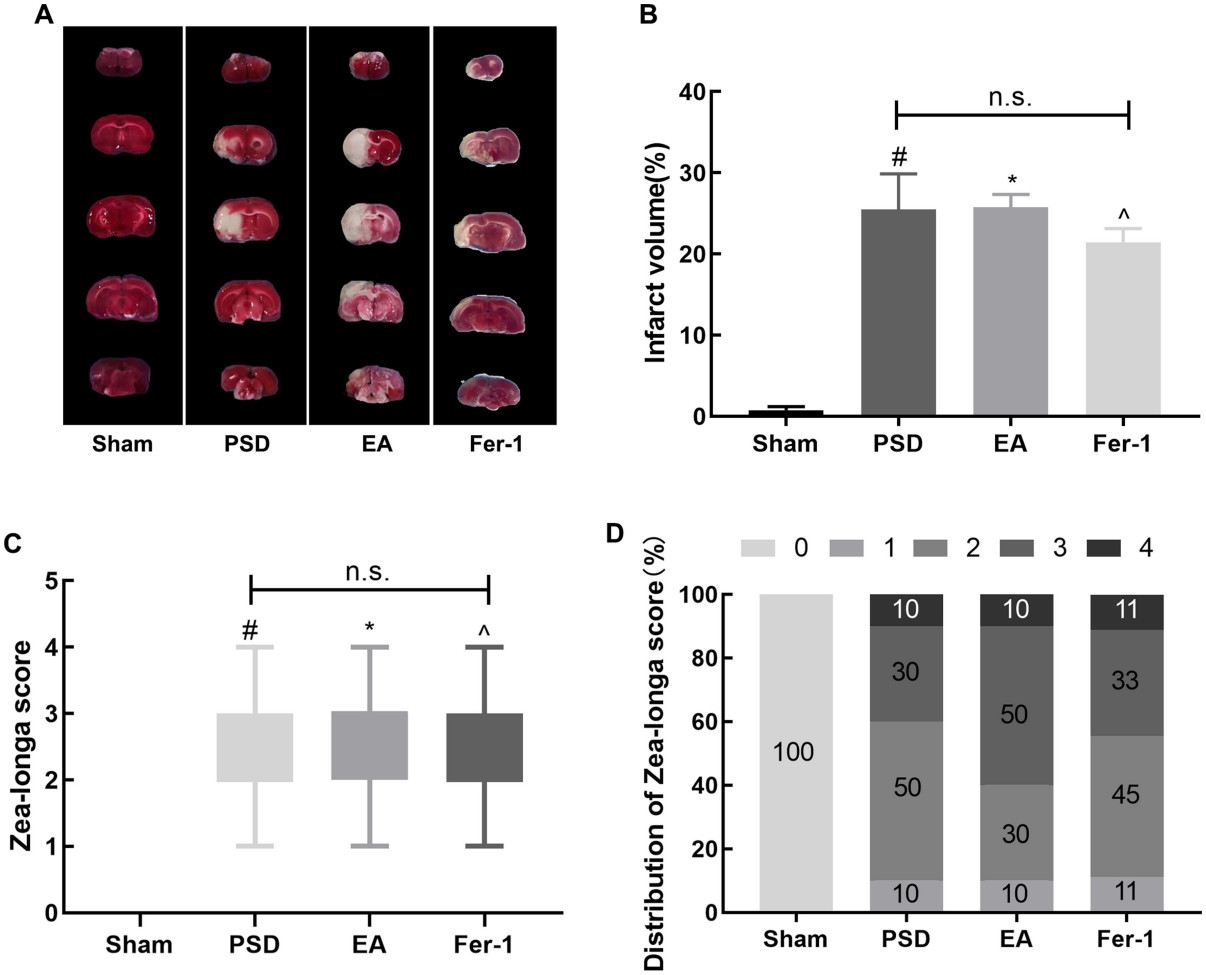
Cerebral infarct volume and neurological scores were increased in rats after MCAO. **(A)** TTC staining. **(B)** Quantitative analysis of infarct volume. **(C)** Zea-Longa score. **(D)** Distribution of Zea-Longa scores (Sham vs. PSD, ^#^*p* < 0.05; Sham vs. EA, ^*^p < 0.05; Sham vs. Fer-1, ^*p* < 0.05).

**Figure 4:**
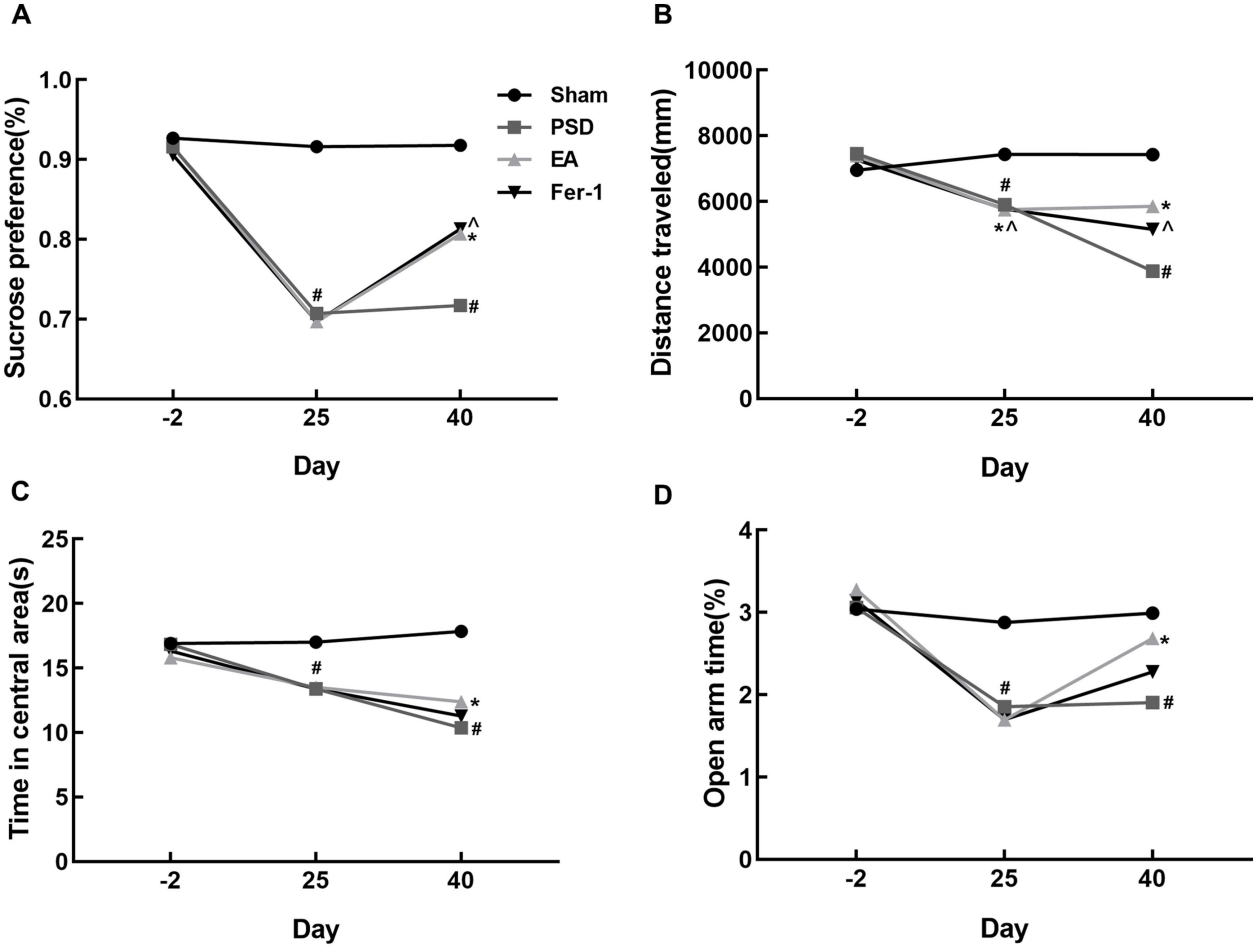
Effects of EA treatment in PSD rats. **(A)** Sucrose preference test. **(B,C)** Distance traveled and time spent in the central area of the open-field test. **(D)** Open arm time of the elevated plus maze test (Sham vs. PSD, ^#^*p* < 0.05; PSD vs. EA, ^*^p < 0.05; PSD vs. Fer-1, ^*p* < 0.05).

As shown in Figure 3, the infarct volume and Zea-Longa scores were significantly increased in the PSD, EA, and Fer-1 groups compared to the sham group, confirming the successful construction of the MCAO model (Figures 3A–D). Figure 4 illustrates that 2 days before MCAO, there were no significant differences in SPT, OFT, and EPMT results among the four groups. However, 25 days after MCAO (following 21 days of CUMS), significant differences in these behavioral tests emerged among the groups. Rats in the PSD, EA, and Fer-1 group displayed depressive symptoms, evidenced by reduced sucrose preference rates, shorter distances traveled, less time spent in the central area, and decreased open arm time, compared to the sham group (all *p* < 0.05). Compared with the PSD group, there were no significance differences in SPT, OFT, and EPMT in rats of EA group or Fer-1 group before treatment (all *p* > 0.05). After 14 days of EA treatment, rats in the EA group showed a significant increase in sucrose preference rate, distance traveled, time spent in the central area, and open arm time compared to the PSD group (all *p* < 0.05). Similarly, rats in the Fer-1 group exhibited significant improvements in sucrose preference rate and distance traveled compared to the PSD group (all *p* < 0.05), but there were no significant changes in the time spent in the central area or open arm time (all *p* > 0.05). No significant differences were observed between the EA and Fer-1 groups in SPT, OFT, and EPMT results (all *p* > 0.05). These findings suggest that both EA treatment and Fer-1 administration exert significant antidepressant effects on PSD rats (Figures 4A–D).

### 3.2 EA treatment ameliorated damage in neurons of prefrontal cortex

**Figure 5:**
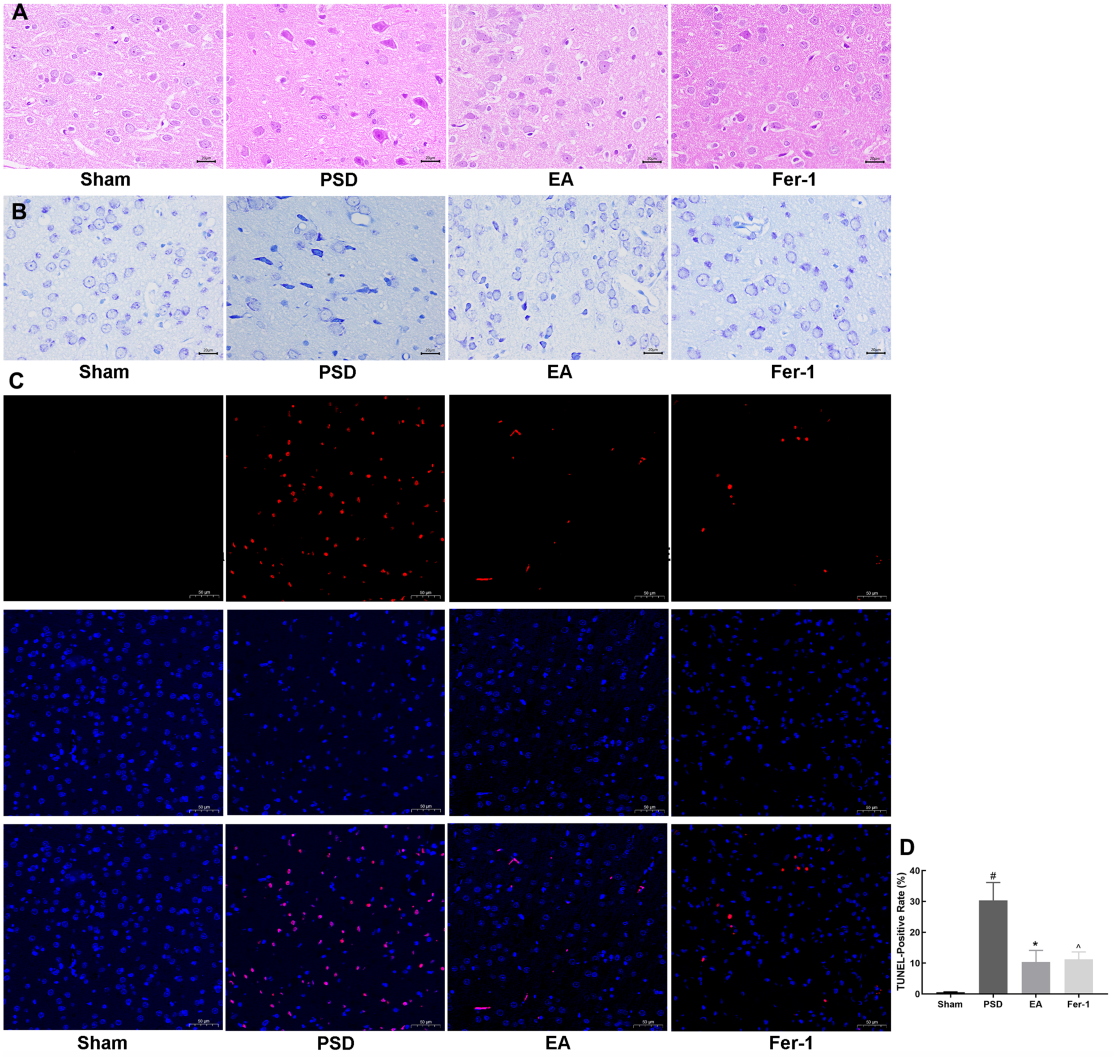
Effects of EA treatment in prefrontal cortex neurons. **(A)** HE staining of prefrontal cortex neurons (scale bar = 50 μm). **(B)** Nissl staining of prefrontal neurons (scale bar = 50 μm). **(C)** Representative fluorescent images of TUNEL-labeled cells in the prefrontal cortex of each group (scale bar = 100 μm). **(D)** TUNEL-Positive rates (Sham vs. PSD, ^#^*p* < 0.05; PSD vs. EA, ^*^p < 0.05; PSD vs. Fer-1, ^ *p* < 0.05).

To observe the morphological changes of neurons in the prefrontal cortex of rats in each group, we used HE and Nissl staining. As shown in Figure 5A, neurons in the prefrontal cortex of rats in the sham group were closely aligned, morphologically intact, and structurally normal. In contrast, after the PSD procedure, neurons exhibited blurred contours, loose arrangements, and a high degree of loss, denaturation, and necrosis. Both EA treatment and Fer-1 administration partially reversed these damages. The pathological changes observed through Nissl staining in the prefrontal cortex were consistent with those seen in HE staining (Figure 5B). Neurons in the prefrontal cortex of rats in the PSD group had abnormal neural structures and a significant loss of Nissl bodies. However, the structure of neurons in the prefrontal cortex was significantly improved, and the number of Nissl bodies increased both in the EA and Fer-1 group.

Given that neuronal death often follows brain damage, we employed TUNEL staining to assess neuronal apoptosis in the prefrontal cortex across the groups. As depicted in Figures 5C,D, almost no apoptotic neurons were seen in the sham group, whereas the number of apoptotic neurons was significantly increased in the PSD group (*p* < 0.05). Both EA treatment and Fer-1 administration significantly reduced the number of apoptotic neurons compared to the PSD group. The data above demonstrated that EA treatment could ameliorate the neuronal damage and death in the prefrontal cortex of PSD rats, and this effect was similar to that of the ferroptosis inhibitor Fer-1.

### 3.3 Erastin administration further increased depression-like behavior in PSD rats, and EA treatment reduced depression manifestations in the Erastin group

**Figure 6:**
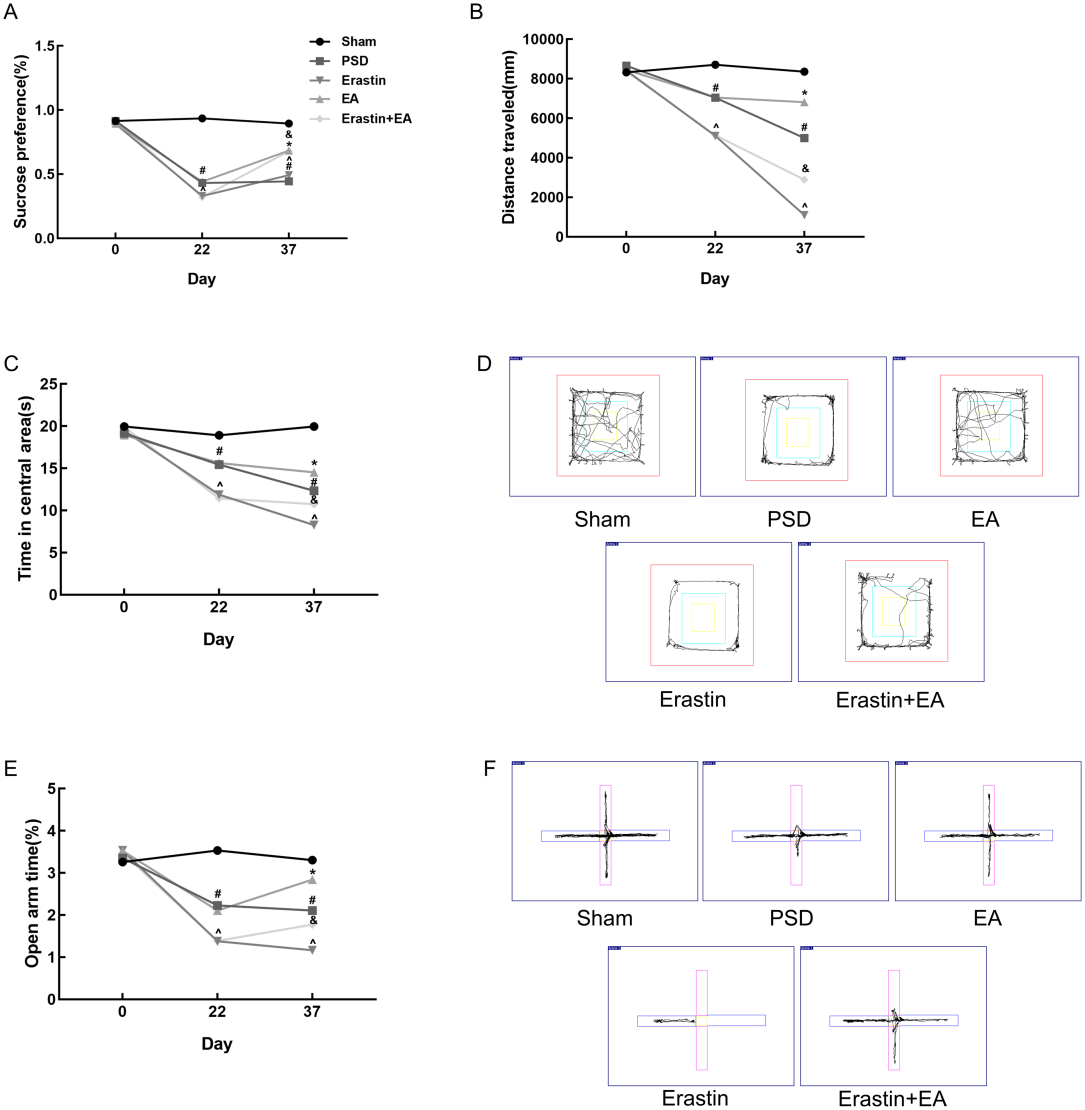
Antidepressant effects of EA treatment after erastin injection. **(A)** Sucrose preference test. **(B,C)** Distance traveled and time spent in the central area of the open-field test. **(D)** Representative trajectory plots of the open-field test. **(E)** Open arm time of the elevated plus maze test. **(F)** Representative trajectory plots of elevated plus maze test. In the images, the horizontal arm is closed and the vertical arm is open (Sham vs. PSD, ^#^*p* < 0.05; PSD vs. EA, ^*^p < 0.05; PSD vs. Erastin, ^^^*p* < 0.05; Erastin vs. Erastin + EA, ^&^*p* < 0.05).

To investigate the mechanism by which EA treatment affects ferroptosis in PSD rats, we administered erastin, a ferroptosis inducer, via injection into the lateral ventricle of PSD rats in both the Erastin and EA + Erastin groups. In the Erastin group, sucrose consumption, activity distance, and exploration of open areas were further reduced compared to the PSD group, indicating that the depression-like behavior in these rats was exacerbated by the ferroptosis inducer. However, EA treatment significantly improved the aforementioned changes (Figures 6A–C,E). These improvements were also observed clearly in the trajectory maps of OFT and EPMT (Figures 6D,F). This suggests that EA treatment alleviated the depression-like behaviors induced by the combination of PSD and erastin, likely through the inhibition of ferroptosis.

### 3.4 EA treatment promoted repair of mitochondrial damage in Erastin rats

**Figure 7:**
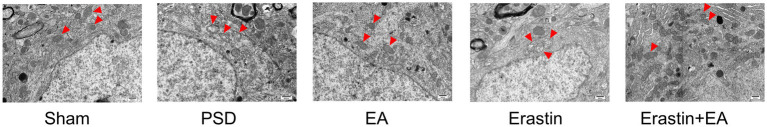
Effect of EA treatment on mitochondrial morphology in prefrontal cortex neurons. Transmission electron microscopy shows the mitochondrial morphology in neurons (scale bar = 500 nm).

Ferroptosis is mainly manifested as morphological changes in mitochondria, and TEM was used to observe the mitochondrial structure inside rat neurons in each group (Figure 7). The structure of the mitochondrial membrane and cristae structures were normal in the sham group. In contrast, mitochondria in the PSD group showed reduced volume, blurred membrane structure, and reduced or absent cristae. EA treatment partially improved these changes. After erastin administration, mitochondrial damage was further aggravated with notable features including mitochondrial swelling, variable shapes and sizes, outer membrane fragmentation, and cristae disappearance. EA treatment in the Erastin group ameliorated mitochondrial damage, as evidenced by the increased number of cristae and improved structural integrity of the mitochondria.

### 3.5 EA treatment reduced iron deposition in Erastin rats

**Figure 8:**
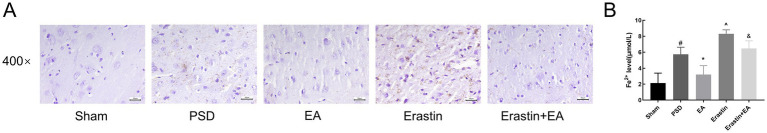
Effect of EA treatment on iron deposition in prefrontal cortex neurons and Fe^2+^ content. **(A)** Perl’s staining of prefrontal cortex neurons (scale bar = 50 μm). **(B)** The Fe^2+^ content levels (Sham vs. PSD, ^#^*p* < 0.05; PSD vs. EA, ^*^p < 0.05; PSD vs. Erastin, ^^^*p* < 0.05; Erastin vs. Erastin + EA, ^&^*p* < 0.05).

Accumulation of intracellular iron is a typical hallmark of ferroptosis. To assess iron accumulation in the rat prefrontal cortex, we employed Perl’s staining and colorimetric analysis. The results of iron deposition in the prefrontal cortex of rats in each group are shown in Figure 8A, and Fe^2+^ content levels are presented in Figure 8B. Compared with the sham group, significant iron deposition was detected and the Fe^2+^ content levels were increased in PSD rats. However, these changes were reversed by EA treatment. Moreover, iron deposition in the prefrontal cortex and serum Fe^2+^ content levels were further increased in the Erastin group, but these increases were also mitigated following EA treatment.

### 3.6 EA treatment restored redox imbalance in Erastin rats

**Figure 9:**
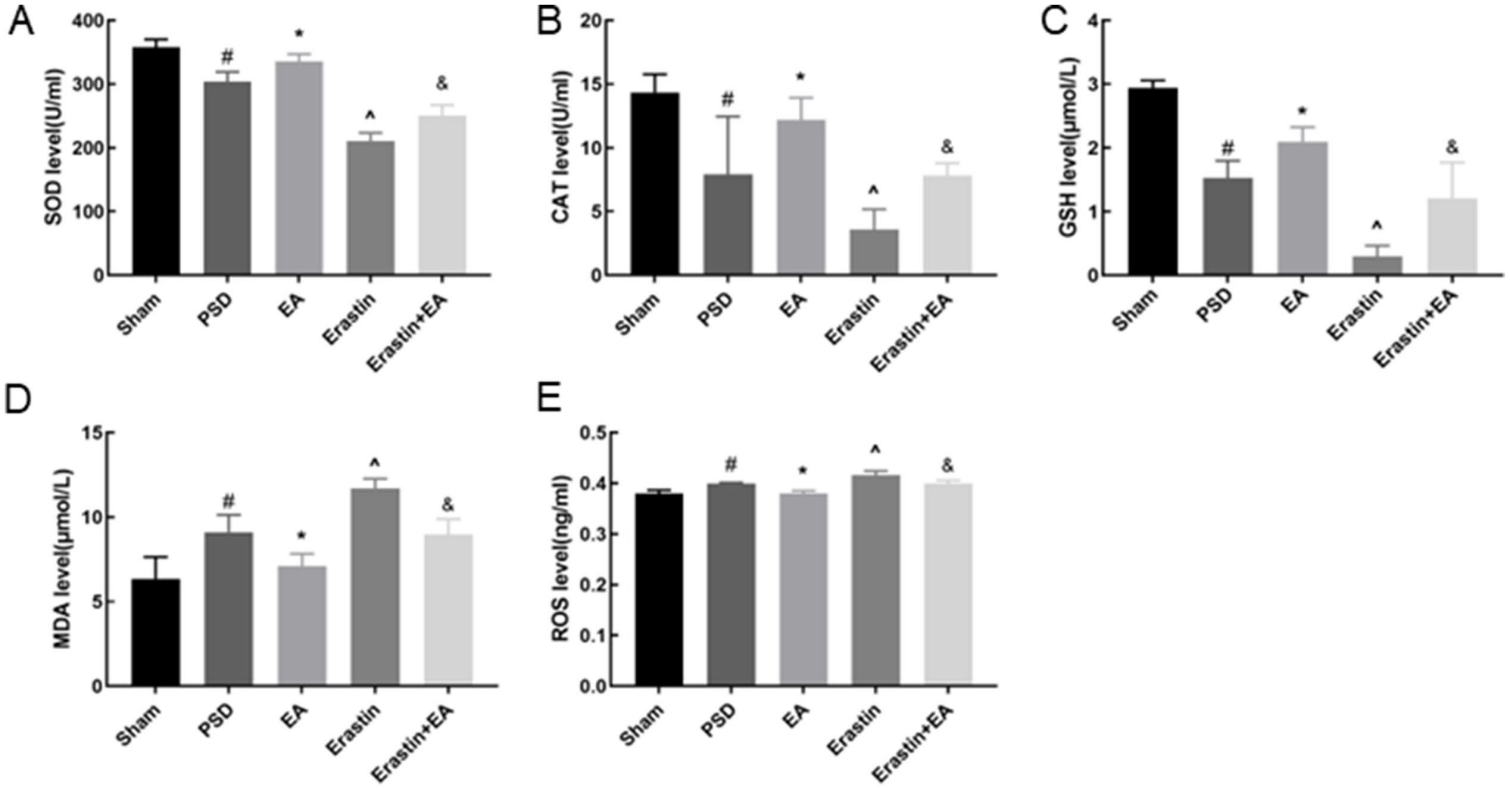
Effects of EA treatment on oxidative stress level. T-SOD, CAT, GSH, MDA and ROS levels **(A–E)** (Sham vs. PSD, ^#^*p* < 0.05; PSD vs. EA, ^*^p < 0.05; PSD vs. Erastin, ^^^*p* < 0.05; Erastin vs. Erastin + EA, ^&^*p* < 0.05).

Ferroptosis is associated with the accumulation of ferrous ions, which increase the level of oxidative stress. Therefore, we used a colorimetric assay and ELISA to detect the indicators of oxidative stress in the rat prefrontal cortex (Figures 9A–E). After the CUMS procedure, the levels of MDA and ROS in the PSD group were increased, whereas the levels of T-SOD, CAT, and GSH were reduced. After erastin administration, changes in the abovementioned indicators were further exacerbated, whereas these changes were reversed by EA treatment.

### 3.7 EA treatment inhibited ferroptosis in Erastin rats

**Figure 10:**
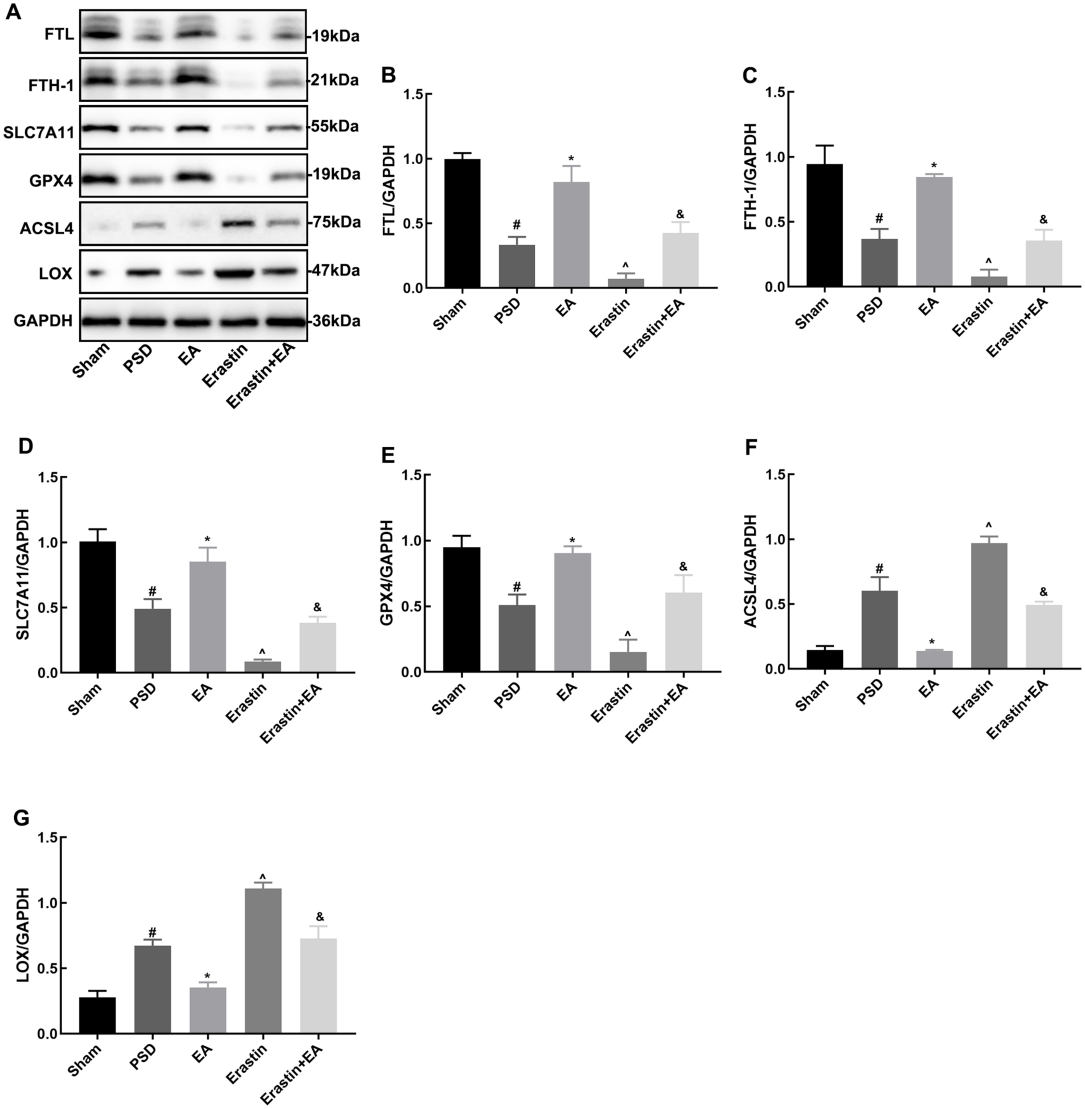
Effects of EA treatment on the expressions of FTH-1, FTL, SLC7A11, GP4, ACSL4, and LOX after erastin injection. Expression levels of FTH-1, FTL, SLC7A11, GPX4, ACSL4, and LOX in the prefrontal cortical tissues **(A–G)** (Sham vs. PSD, ^#^*p* < 0.05; PSD vs. EA, ^*^p < 0.05; PSD vs. Erastin, ^^^*p* < 0.05; Erastin vs. Erastin + EA, ^&^*p* < 0.05).

Ferroptosis is accompanied by significant alterations in the expression of proteins related to iron metabolism, the glutathione system, and lipid metabolism. To investigate these alterations, WB was used to detect the expression of FTH-1, FTL, SLC7A11, GPX4, ACSL4, and LOX in the rat prefrontal cortex (Figures 10A–G). In the PSD group, compared to the sham group, there was a significant decrease in the expression of SLC7A11, GPX4, FTH-1, and FTL proteins, while the expression of ACSL4 and LOX proteins was significantly increased. These protein expression changes were further exacerbated following erastin administration. However, EA improved these changes in the PSD and Erastin groups.

## 4 Discussion

PSD is a common complication after a stroke, significantly impacting the recovery of neurological functions and the ability of patients to reintegrate into society (Medeiros et al., 2020). However, the pathophysiology of PSD remains largely unclear, which hinders the development of novel antidepressant treatment strategies (Castilla-Guerra et al., 2020). Therefore, there is an urgent need to understand the mechanisms underlying PSD and to explore new and effective treatments for this disease. In this study, we used a combination of MCAO and CUMS to construct PSD model rats. The model rats were then treated with electroacupuncture (EA) for 14 days to investigate its antidepressant mechanisms. Our results revealed that EA significantly reduced neurological deficits, increased sucrose consumption, enhanced spontaneous activity, and promoted exploratory behavior. Additionally, EA attenuated neuronal damage in the prefrontal cortex of PSD rats, showing effects comparable to the ferroptosis inhibitor Fer-1. In addition, EA ameliorated the exacerbation of depression-like behavior induced by ferroptosis inducer erastin and inhibited the ferroptosis in neurons of prefrontal cortex in PSD rats.

### 4.1 Repair of prefrontal cortex neurons mediated the antidepressant effect of EA in PSD rats

Prefrontal cortex is one of the most severely damaged brain regions in individuals with major depressive disorder (Pizzagalli and Roberts, 2022). Structural and functional abnormalities of the prefrontal cortex are closely related to depression-like behaviors, such as negative processing bias, anhedonia, and learned helplessness (Pizzagalli and Roberts, 2022). A meta-analysis of repetitive transcranial magnetic stimulation for the treatment of PSD has demonstrated that targeting the prefrontal cortex yields significant antidepressant effects (Wang T. et al., 2023). Additionally, animal studies have also shown that intranasal drug delivery in PSD rats exerts antidepressant effects primarily by increasing BDNF expression in the prefrontal cortex, without significant changes in the hippocampus (Chen et al., 2020). In this study, we utilized HE and Nissl staining to observe the structure of the prefrontal cortex in rats. Our results indicated that PSD modeling led to severe brain tissue edema, loosening of the nerve fiber network, widespread neuronal degeneration and necrosis, and a reduction in the number of Nissl bodies. Notably, significant improvements in the morphological structure of the prefrontal cortex were observed after EA treatment. In addition, EA significantly reduced the increased prefrontal cortex neuronal apoptosis induced by PSD. Given the observed improvements in depression-like behaviors in PSD rats treated with EA, we suggest that the improved structure and function of the prefrontal cortex might underlie the antidepressant effects of EA, which is consistent with our previous studies (Ding et al., 2023). Therefore, the present study further explored the mechanism of action of EA by targeting its effects on the prefrontal cortex.

### 4.2 EA ameliorated depression-like behavior in PSD rats by inhibiting ferroptosis in prefrontal cortex neurons

Ferroptosis, a newly identified type of regulated cell death, has been shown to be associated with neurological disorders, including stroke and depression (Xu et al., 2023; Du et al., 2023). Xu et al. (2022) found that alcohol-treated mice had a significant increase in depression- and anxiety-like behaviors, which may be related to the activation of ferroptosis. Studies have suggested that ferroptosis may emerge as a novel target in the treatment of ischemic stroke (Liu et al., 2023). In addition, multiple animal studies have shown that inhibiting ferroptosis through drug therapy significantly improves depression-like behavior in animals exposed to CUMS (Jiao et al., 2021; Li et al., 2023; Wang X. et al., 2023). This suggests that ferroptosis may be a potential therapeutic target for PSD. In our study, Fer-1, served as the positive control, ameliorated the depressive behaviors and neuronal injury after the PSD modeling. Fer-1 was one of the first synthetic radical-trapping antioxidants reported to block ferroptosis and it is widely used as reference compound (Scarpellini et al., 2023). Previous studies have demonstrated the protective effects of Fer-1 on cerebral ischemia/reperfusion injury (Liu et al., 2023; Shi et al., 2024), consistent with our results. Interestingly, EA treatment produced similar antidepressant and neuroprotective effects as Fer-1. These results led us to hypothesize that EA might alleviate depressive behaviors and neuronal injury in PSD by regulating ferroptosis.

Ferroptosis is distinct from traditional cell death modes, such as apoptosis, autophagy, and necrosis, in terms of cell morphology, biochemical characteristics, and genetic level (Li et al., 2020). Abnormal mitochondrial morphology is a hallmark of ferroptosis, and mitochondria in the brains of CUMS-exposed rats show ultrastructural changes typical of ferroptosis (Zhang et al., 2021). In the present study, we used TEM to observe ultrastructural changes in mitochondria. Following the development of PSD, mitochondria exhibited increased volume and reduced, degraded, or disappeared cristae. The mitochondrial damage was improved with EA. Meanwhile, the combined effect of PSD and erastin exacerbated the abovementioned changes in mitochondria. The production and accumulation of reactive oxygen species (ROS), closely associated with mitochondrial dysfunction, were significantly increased in the prefrontal cortex following PSD and erastin administration (Zorov et al., 2014). EA treatment significantly improved mitochondrial morphology and structure, accompanied by a reduction in ROS levels in the prefrontal cortex.

Iron overload is one of the key factors in the occurrence of ferroptosis. Free extracellular iron ions (Fe^3+^) enter cells and are converted to ferrous ions (Fe^2+^), which are then transferred to the cell cytoplasm. Free Fe^2+^ in the cytoplasm can bind to ferritin and be temporarily stored intracellularly. FTH-1 and FTL are key proteins that regulate cellular iron metabolism in the ferroptosis pathway. Under the action of FTH-1 and FTL, Fe^2+^ is stably stored in cells in a non-toxic manner. However, excessive iron intake can lead to iron overload and induce ferroptosis. Iron overload has been shown to be involved in the pathological process of ischemic stroke (Guo et al., 2023). Furthermore, depression is often associated with disturbances in iron metabolism (Oliveira et al., 2017). Previous studies have found that the use of the iron chelator deferiprone increased locomotor activity and decreased the latency to ingest in depressed mice, presenting a significant antidepressant effect (Uzungil et al., 2022). Here, we examined iron levels in the prefrontal cortex and found that PSD led to increased ferrous iron content and abnormal iron deposition in this brain region. Erastin administration further exacerbated these changes, whereas EA effectively ameliorated them. Meanwhile, we also detected the protein expressions of FTH-1 and FTL using WB. FTH-1 and FTL expressions were significantly reduced in the PSD group, which was further aggravated in the Erastin group. It suggests that the combined action of PSD and erastin promotes iron overload, and EA treatment was beneficial to restore the balance of iron metabolism.

Another key process triggered by ferroptosis is lipid peroxidation within cells. Previous studies on stroke and depression have demonstrated that intracellular Fe^2+^ overload contributes to the synthesis of lipoxygenase (LOX) and the production of lipid peroxides, which in turn induces ferroptosis (Zhou et al., 2021; Zuo et al., 2022). ACSL4 and LOX are important proteins that regulate the lipid peroxidation process. Li et al. (2023) treated CUMS mice with ferrostatin-1, an inhibitor of ferroptosis, and found reduced expression levels of ROS, ACSL4, and LOX, which is consistent with our findings. This suggests that EA inhibits ferroptosis by reducing lipid peroxidation.

Insufficient anti-lipid peroxidation capacity of System XC- and GPX4 enzymes accelerates the onset of ferroptosis. In ferroptosis, System XC- and GPX4 are jointly responsible for scavenging lipid peroxides. SLC7A11 is a key protein constituting System XC- that facilitates the uptake of extracellular cystine and the release of glutamate. This process promotes GSH synthesis, protects cells from oxidative stress, maintains cellular redox balance, and prevents cell death due to lipid peroxidation (Yuan et al., 2022). A growing body of research suggests that stroke often have functional inhibition of systems XC- and GPX4, decreased GSH, and increased oxidative stress (Hu et al., 2022; Liu et al., 2023). However, the relationship between depression and System XC^−^ remains underexplored. In our study, we used WB, colorimetric assay, and ELISA to detect the function of the antioxidant system in rats. We found that PSD led to decreased expressions of SLC7A11 and GPX4, along with reduced levels of GSH, catalase (CAT), and superoxide dismutase (SOD), and increased levels of malondialdehyde (MDA). These changes were further exacerbated by erastin administration. However, EA significantly ameliorated the abovementioned changes, demonstrating antioxidant effects.

To date, this is the first study to investigate whether ferroptosis is involved in the pathological process of PSD. Our findings demonstrated that EA can reduce neurological deficits and enhance the spontaneous activity and exploration behavior of rats. In addition, EA could inhibit prefrontal cortex neuronal ferroptosis by reducing iron deposition, decreasing lipid peroxidation, and enhancing antioxidation. The use of erastin, a ferroptosis inducer, further highlighted the inhibitory effects of EA on ferroptosis, suggesting the importance of considering erastin as a reagent control in such studies. This study provides theoretical evidence at the molecular level that EA is an effective rehabilitation strategy for PSD.

## Data Availability

The original contributions presented in the study are included in the article/Supplementary material, further inquiries can be directed to the corresponding authors.
