# Supplementary material for: Electroacupuncture ameliorates depression-like behaviors in rats with post-stroke depression by inhibiting ferroptosis in the prefrontal cortex
Source: Front Neurosci. 2024 Oct 3;18:1422638. doi: 10.3389/fnins.2024.1422638 (PMC11483888; doi:10.3389/fnins.2024.1422638)
Supplement: Supplementary file 1 [file Data_Sheet_1.pdf]

10:54

Zheng Xinyue

April 11, 2023 20:49 PM

Billing Unit: Henan University of Chinese Medicine

Contact: Feng Yixuan

Tel: 15649683028

Strain: SD rat

Gender: Male

Quantity: 75 pieces

Age or weight: 220-240 g

Mode of transport: carton transport

Delivery address: Jinshui District, Zhengzhou City, Henan Province

South University of Chinese Medicine Longzihu Campus

Sister this is some of the information we ordered before you can refer to the order ~

Okay, when do we order rats?

We can do it now. We have to wait in line at the animal center.

On that online system?

Apply on there and then contact the delivery mouse to place the order into the mouse quickly

So Ronon and I will do it now?

Yeah, I think the Animal Center line is online now. It's not like we used to.

扫描全能王 创建

10:46 45

Yu Mingyue

Gotta read his manual.

This, I guess, should be measured overall.

If it was ferrous, he would mark the ferrous ion.

Oh ~ Souga

Oh yeah ~

Sister like this kind of test kit to buy a box is enough to test 5 groups of it

Right

He'll split 48 and 96.

48t is his reagent is enough to measure 48 holes, 96t is able to measure 96 holes.

Is it better to buy more holes?

Can buy 96 holes.

Can be repeated several times

扫描全能王 创建

Sister total iron ion / ferrous ion colorimetric kit What is the difference between these two?

The total iron ion kit measures ferrous and trivalent iron, and ferrous ion measures ferrous.

What I'm looking at is ferrous ions, which produce the Fenton reaction, which causes lipid peroxidation.

Oh, oh, I was wondering about the iron death process. What kind of ion should I test?

ACE: What I'm looking at is ferrous ions, which produce the Fenton reaction, which causes lipid peroxidation.

Uh-huh.

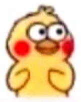

I searched the Ivory Treasure. It's only Soleil and it's called Tissue Iron Test Kit.

It seems that Teacher Yao didn't put this product on the ivory bag

品

Yeah, I think so.

Is it true that a name like Shijie is always measured?

扫描全能王 创建

10:45

Yu Mingyue

February 6, 2023 15:09 PM

What brand did you buy for iron ion kit?

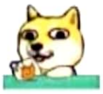

Elieret

Oh, okay.

elabscience

Did you buy it at the school ivory?

No, I got it from Miss Yao.

OK~

This should be able to search, you can try

Small joy: is in the school ivory treasure to buy it

Good drop.

I'll try.

February 6, 2023 15: 11 PM

扫描全能王 创建

10:53

Wu Ruonan, Grade 22

Sister, I think the dilution concentration is a range, how much do we usually use

1: 1000?

Mar 13, 16: 32 PM

Usually start with the smallest concentration.

中

If the concentration is not good, then slowly increase it.

I think the top is 1: 1000.

I just looked at the one you posted, and there is no 1: 1000.

🤣

My picture is according to the instructions

The instructions are all a range

Have to debug one by oneself

If you can get the results, press the dilution concentration.

行

扫描全能王 创建

10:53

Wu Ruonan, Grade 22

What are the concentrations of these antibodies? SLC7A11⊠GPx4⊠ ACSL4, LPCAT3), LOX

Mar 13, 16: 18 PM

I'll shoot you.

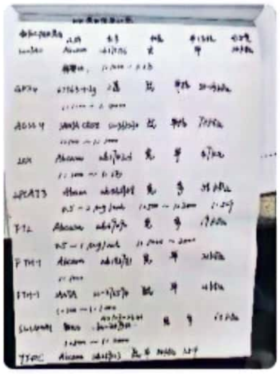

Do you use these brands too?

Then I don't know.

I used these.

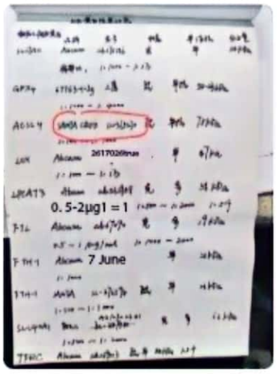

Doesn't this have a Chinese name?

扫描全能王 创建

10:53 44

Wu Ruonan, Grade 22

I would like to ask the ELISA data is arranged like this? Is the amount of data in each group different?

组 A	Group B	组 C	组 D
Normal group, sham operation group, model group			Electroacupuncts
1.34066	1.02764	0.27935	0.84207
1.82037	1.51959	0.52962	0.30549
1.20489	0.72461	0.39297	1.22922
0.84913	1.35462	0.53341	0.94043
0.91592	1.29185	0.13481	0.55602
		0.28375	0.60074
		0.04830	0.42900
			0.52203
			0.63023
			0.88758

Mar 9, 16: 24 PM

The amount of data in each group must be the same

Right?

I feel like this is all wrong.

😂

Well, you didn't do this?

The lowest three?

No

Elder sister

扫描全能王 创建

10:52

Wu Ruonan, Grade 22

Excuse me, where did you buy the GSH and MDA test kit?

All the brands I buy are Eli Reiter's.

好

This is the brand that most kits use.

^ ^: All the brands I bought were Eli Reiter's.

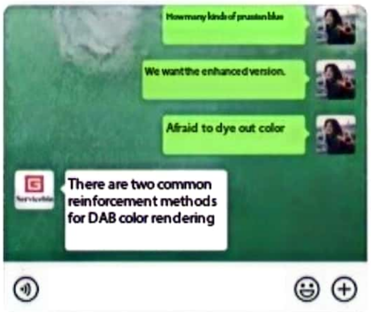

Probably the second one. 🤔

好

^ _ ^: Usually the kit uses this brand.

对

好

I think I'm gonna take a shower. Last night I broke out in a sweat. My hair is even greyer.

But you just got better. 🫣

扫描全能王 创建

10:52

Wu Ruonan, Grade 22

January 16, 21: 06 pm

Sister, HE dyeing, Prussian blue dyeing need to buy what? I bought one in advance.

下

Jan 16, 21: 13 PM

Buy the corresponding dyeing kit, sister

From Tianchi?

You can buy it through Tianchi

But you can check the signs yourself.

好

I remember the prussian blue better get the enhanced version

Sister, do you recommend this?

^ _ ^: I remember the Prussian blue better buy the enhanced version

Before, Brother Qi Shikui said that Seville's enhanced version can be dyed

^ ^: I remember the Prussian blue better buy the enhanced version

The regular version won't dye out

扫描全能王 创建

10:51

Wu Ruonan, Grade 22

It should be.

You try.

Is the percentage wrong? I remember when my graph came out, the ordinate was 100

Ww: The percentage of this coming out is not 100

I'll try again.

Jan 7, 23: 42 PM

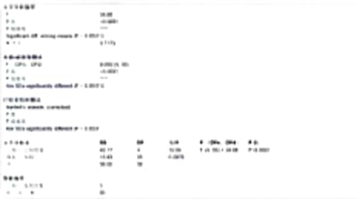

Excuse me for a moment

Does this even make sense?

Can P be less than 0.05?

I think it's 0.0001.

This is not the result of multiple comparisons.

Comparison between groups

[illegible]

扫描全能王 创建

10:51

Wu Ruonan, Grade 22

7 Jan 23: 12 pm

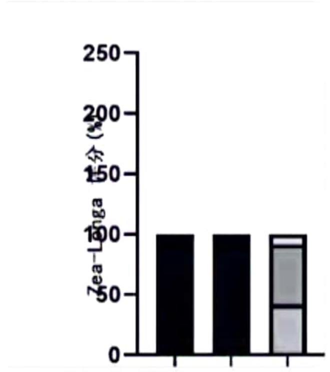

Elder sister

I just tried it.

The percentage of this coming out is not 100

😭

What went wrong?

😭

The ordinate value on the left is adjustable.

Double click to adjust its range

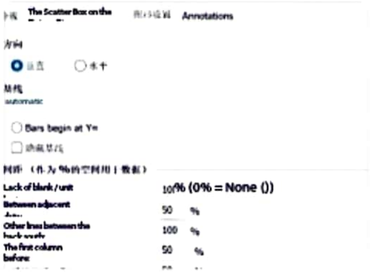

扫描全能王 创建

11:10

Tang Yuchuang

Nov 2, 2023, 20: 33 PM

Hello, brother.

Free time can be in the public under some post-stroke depression, iron death related literature to look at the first to understand

https://pubmed.ncbi.nlm.nih.gov/

This is a public URL.

Post Stroke Depression Search Post-stroke depression on the line Iron death search Iron droop

All right, sister.

Is this a related experiment this week?

English literature with translation software (Zhiyun literature translation or little green whale) can see

The last time I showed you

OK 👌

You may be involved in the paper writing later, so get to know it in advance

扫描全能王 创建

11:10

唐玉创

2023年11月2日 晚上20:33

哈喽 师弟

有空的时候可以在公众场合下一些卒中后抑郁、铁死亡相关的文献看看先了解了解

https://pubmed.ncbi.nlm.nih.gov/

这是公开的网址

卒中后抑郁搜卒中后抑郁症就行铁死亡搜铁下垂

好的，师姐

这周是要做相关的实验吗

英文文献用翻译软件 （知云文献翻译 或者小绿鲸）可以看

上次让你们看的就是

好的👌

后面可能会让你们参与论文写作所以先提前了解了解

扫描全能王 创建

11:10

Tang Yuchuang

October 24, 2023 21: 15 PM

I'm Tang Yuchuang

This is the content of the greeting

You have added the building height to your home, now you can start chatting.

Go directly to the second floor of Junxue Building tomorrow
行

All right, sister.

Do you need a lab coat tomorrow?

Don't bring it yet

OK

Do you know anything about WB?

Sister Wu sent us a video

Let's watch the video and learn.

OK. Okay.

October 25, 2023 11: 37 AM

扫描全能王 创建

11:10

Qin Yanyun

November 2, 2023, 20: 28 pm

When the younger sister is free, you can look at some post-stroke depression and iron death related literature under pubmed to understand first

Okay, thank you.

That's nice.

😂

https://pubmed.ncbi.nlm.nih.gov/

This is the website of pubmed

Sister knows me

Post stroke depression, iron death, ferroptosis

I have never used pubmed.

This literature isn't mostly in English, is it?

After all, it might be a little early for you.

Yeah, yeah, it's all in English.

扫描全能王 创建

11:10 39

Qin Yanyun

October 25, 2023 11: 37 AM

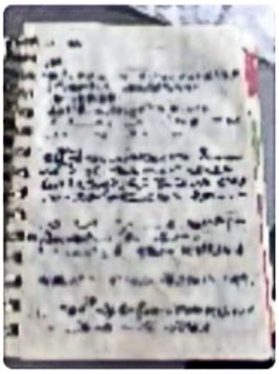
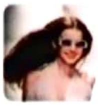

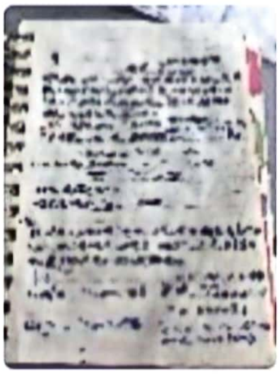
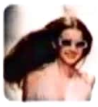

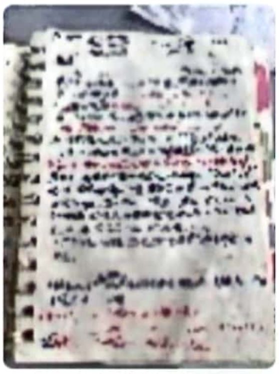
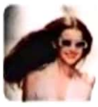

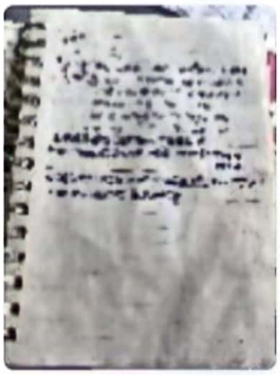
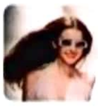

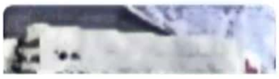
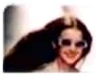

扫描全能王 创建

10:47

Brother Yan Qi Shikui

December 18, 2022 10: 22 AM

Okay, what kind of results do you need? I'll come back tonight and find you.

December 18, 2022 10: 33 AM

Ok brother I need the results of apoptosis, prussian blue stain and iron kit

Please help me find it tonight 🌞

Can I get the results of WB / ELISA?

We can.

好

Didn't the results of apoptosis come out?

No iron kit. I didn't do it. You can ask the moon. He did this.

Okay, I'll check with you later.

Mm-hmm.

扫描全能王 创建

10:44

Ken - Gao Jing

All right, sister.

Gao Jing: This still needs to be changed

I'm sure I'll have to change it.

Dec 23, 2023, 16: 59 PM

I'll get in touch and see how it's polished.

Dec 23, 2023 17: 02 PM

All right, sister.

No problem, we are not native English, do you have a Chinese manuscript?

Or written directly?

Dec 23, 2023 17: 11 PM

There is no Chinese script sister

It is written directly in English and some used deepl translation

OK

12-25-2023, 14: 41 PM

扫描全能王 创建

10:44 45

Ken - Gao Jing

Dec 23, 2023, 16: 08 PM

So I put the track map of mine and the overhead map together. Tunel map is in fig2 with the other map.

Gao Jing: Open Field Experiment and Maze Trail Map don't put together, open field put together, maze put together than

Discussion I summarized the results of the experiment in the third paragraph there and the preface is not the same as I see Zhimin's article discussion part is also introduced PSD, I think I can not get around PSD, so I briefly introduced it.

It took me a week to write every day I was almost blind 😂

Dec 23, 2023, 16: 23 PM

OK

This still needs to be changed

^ _ ^: Sister similar statistics together look nice and neat, so I put the mine and elevated track map...

You ask Zhimin's opinion

Dec 23, 2023, 16: 32 PM

[illegible]

扫描全能王 创建

10:44

Ken - Gao Jing

December 23, 2023 08: 55 AM

Open field experiment and the maze track map do not put together, open field put together, the maze put together more appropriate

The general plan of the tunnel is also, and his representative picture is placed in a figure.

December 23, 2023 09: 03 AM

Always feel a lot of words are not appropriate

😭

Is it writing too fast?

Not particularly authentic.

December 23, 2023 09: 06 AM

Discussion 写作思路:
The first paragraph summarizes the results of the experiment. Do not write induction and discuss the results of the experiment

Can be written in a logical order from macro to micro

Ethology - Pathology - Molecular Biology

扫描全能王 创建

10:43

Ken - Gao Jing

Dec 21, 2023 12: 46 PM

English - drafts.docx
101.0 KB
W

WeChat Computer Version

Illustrations and explanations. docx
77.7 MB
W

WeChat Computer Version

Sister, the article changed when free to help look at it

OK

Dec 22, 2023 16: 49 PM

How did you write this time?

Dec 22, 2023 16: 57 PM

I'm a little busy these two days. I'll see one tomorrow morning.

下

OK

I hope you will see more changes and suggestions. 🌹

[illegible]

扫描全能王 创建

10:43

Ken - Gao Jing

Is there any need to change the details of the direct first touch up?

Yeah.

To change

I'll go to school tomorrow and tell you exactly

I'll change it.

OK

December 12, 2023 06: 54 AM

Yi Xuan, change your paper in the lab this morning

Around 9: 30.

December 12, 2023 07: 13 AM

All right, sister.

December 12, 2023 09: 26 AM

Coming over?

12 / 12 / 2023 09: 38 AM

扫描全能王 创建

10:43

Ken - Gao Jing

December 6, 2023 11: 28 AM

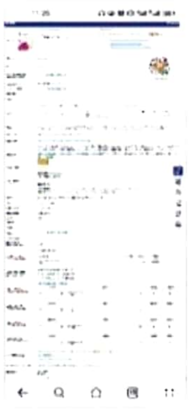

See how much work he does.

^_^:

Look for articles with similar themes.

OK

December 6, 2023 11: 40 AM

Feel to touch up

I borrow Mr. Wang Mengyu first run it

We'll pay for it in January and we'll save some polish.

费

Good language is bad.

Do you need to change the details directly?

扫描全能王 创建

10:42

Ken - Gao Jing

December 6, 2023 11: 19 AM

Frontiers in Neuroscience

Check this magazine.

December 6, 2023 11: 25 AM

Impact Factor | Impact Factor Prediction of Traditional Chinese Medicine Journals at the End of 2023 - General Practice and Replenishment

Traditional Chinese Medicine Department, Official Account template of Traditional Chinese Medicine.

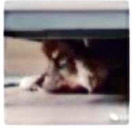

Chinese and western scientific research

December 6, 2023 11: 28 AM

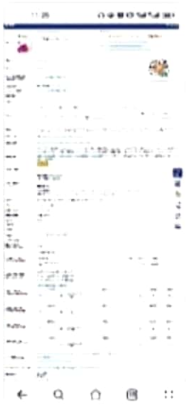

See how much work he does.

^_^:

Look for articles with similar themes.

扫描全能王 创建

10:43

Ken - Gao Jing

Dec 21, 2023 12: 46 PM

English - drafts.docx
101.0 KB
W

WeChat Computer Version

Illustrations and explanations. docx
77.7 MB
W

WeChat Computer Version

Sister, the article changed when free to help look at it

OK

Dec 22, 2023 16: 49 PM

How did you write this time?

Dec 22, 2023 16: 57 PM

I'm a little busy these two days. I'll see one tomorrow morning.
下

OK

I hope you will see more changes and suggestions. 🌹

[illegible]

扫描全能王 创建

10:43

Ken - Gao Jing

Is there any need to change the details of the direct first touch up?

Yeah.

To change

I'll go to school tomorrow and tell you exactly

I'll change it.

OK

December 12, 2023 06: 54 AM

Yi Xuan, change your paper in the lab this morning

Around 9: 30.

December 12, 2023 07: 13 AM

All right, sister.

December 12, 2023 09: 26 AM

Coming over?

12 / 12 / 2023 09: 38 AM

扫描全能王 创建

10:43

Ken - Gao Jing

December 6, 2023 11: 28 AM

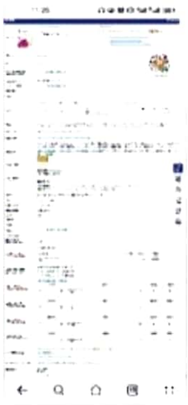

See how much work he does.

^_^:

Look for articles with similar themes.

OK

December 6, 2023 11: 40 AM

Feel to touch up

I borrow Mr. Wang Mengyu first run it

We'll pay for it in January and we'll save some polish.

费

Good language is bad.

Do you need to change the details directly?

扫描全能王 创建

10:42

Ken - Gao Jing

December 6, 2023 11: 19 AM

Frontiers in Neuroscience

Check this magazine.

December 6, 2023 11: 25 AM

Impact Factor | Impact Factor Prediction of Traditional Chinese Medicine Journals at the End of 2023 - General Practice and Replenishment

Traditional Chinese Medicine Department, Official Account template of Traditional Chinese Medicine.

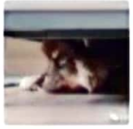

Chinese and western scientific research

December 6, 2023 11: 28 AM

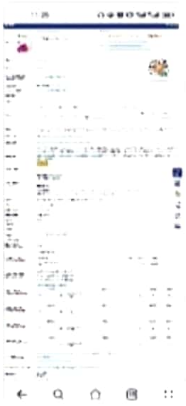

See how much work he does.

^_^:

Look for articles with similar themes.

扫描全能王 创建

The thesis is finished and I will send it to you for help

English - drafts.docx
97.4 KB

W

WeChat Computer Version

Illustrations and explanations. docx

91.3 MB

W

WeChat Computer Version

Dec 4, 2023 15: 14 PM

好

Dec 5, 2023 14: 14 PM

6"

Is Li Xuan ready to vote for which journal?

6"

I have a good two days to change, should need to polish.

扫描全能王 创建

English - drafts.docx

2.0 MB

WeChat Computer Version

扫描全能王 创建

manuscript(4).docx
101.9 KB

WeChat Computer Version

This is the format, but the content has not changed to see that OK

July 21, 2023 10: 20 AM

Manuscript - copy.docx
110.3 KB

WeChat Computer Version

Okay, I just changed that.

July 21, 2023, 11: 24 AM

manuscript(5).docx
107.1 KB

WeChat Computer Version

I'm done with this, too. Press this. Come on. Don't look at the top.

OK

扫描全能王 创建

10:57 43

Ken Ting Chi-min

12 / 7 / 2023 17: 16 PM

How do you get it to warm up first? I've set it to 37 degrees.

The temperature is still 24.6, not rising.

If you set 37, it should slowly go up.

For a while, nothing changed.

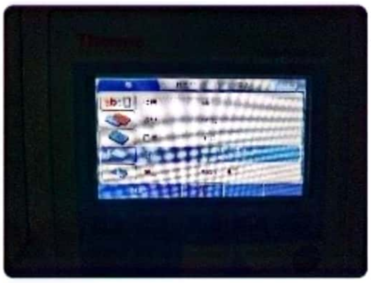

Your board is in!

?

No

You click on the start should remind you that the temperature is not

And then it will heat up.

That reminds me, but the temperature isn't rising.

[illegible]

扫描全能王 创建
